# Supplementary material for: Facile Synthesis of Microporous Ferrocenyl Polymers Photocatalyst for Degradation of Cationic Dye
Source: Polymers (Basel). 2022 May 6;14(9):1900. doi: 10.3390/polym14091900 (PMC9100194; doi:10.3390/polym14091900)
Supplement: Supplementary file 1 [file polymers-14-01900-s001.zip › polymers-1653606-supplementary.pdf]

Supplementary Materials

Facile synthesis of microporous ferrocenyl polymers photocatalyst for degradation of cationic dye

Bing Zhang, Zhiqiang Tan, Yinhu Zhang, Qingquan Liu*, Qianxia Li, Gen Li*

School of Materials Science and Engineering, Hunan Provincial Key Laboratory of Advanced Materials for New Energy Storage and Conversion, Hunan University of Science and Technology, Taoyuan Street, Xiangtan, 411201, PR China

* Correspondence: qqliu@hnust.edu.cn (Q.L.); genKevinli@hnust.edu.cn (G.L.)

Experimental Section

Materials

Carbazole， pyrrole，1,1'-ferrocenedicarboxaldehyde，methylene blue，cupric sulfate anhydrous，edetate disodium，tert-butyl alcohol and L-histidine purchased from Shanghai Aladdin Bio-Chem Technology Co. LTD. and used without further purification. 1,4-Dioxane was purified by dehydration with 5A molecular sieves and distillation under reduced pressure prior to use. Other reagents are used as received.

Instrumentation

FT-IR spectra were collected in transmission mode in KBr pellets at room temperature on a FTIR-2000 spectrometer (Perkin-Elmer in America). Powder X-ray diffraction (PXRD) patterns were recorded on Rigaku Smartlab 9KW diffractometer for Cu Kα radiation (α = 1.5406 Å, with a step size of 0.02° in 2θ from 3° to 30°. Thermogravimetric analysis (TGA) curves were recorded on a Mettler GA/SDTA851 thermogravimetric analysis instrument by heating the samples up to 800 °C with ramping rate of 10 °C min^{-1} under nitrogen flow. Morphology of the samples was observed by scanning electron microscopy (SEM, JSM-6380 LV). The X-ray photoelectron spectroscopy (XPS) experiments were performed on a K-Alpha 1063 spectrometer (Thermo Fisher Scientific) and the core level spectra were measured using a monochromatic Al Kα X-ray source (hv = 1386.6 eV). Nitrogen sorption porosimetry was performed on a Micromeritics ASAP 2020 volumetric adsorption analyzer. The experiments were carried out at the temperature of liquid nitrogen (77.3 K).The samples were first heated in a tube under vacuum at 120°C for 20 h to remove adsorbed materials. Pore size distribution curves and the pore volumes of MOPs were derived from the adsorption branches of the isotherms using the nonlocal density functional theory (NL-DFT).The UV-vis absorbance spectra were recorded on a UV–vis Spectrophotometer(TU-1901) in a 2mm×10mm cuvette. The photocatalytic degradation was carried out under DS-GHX-V (Dusi Instruments Co., Ltd., Shanghai, China) equipped with an internal xenon lamp and a visible light cut-off filter (λ > 420 nm).

Preparation of the Ferrocenyl Microporous Polymer Networks.

The polymerizations of the two polymer networks were performed in a similar procedure, so only the synthesis details of PFcMOP was described here as an example. Under nitrogen flow, 1,1'-ferrocenedicarboxaldehyde (0.428g, 2 mmol), pyrrole (0.277 mL, 4 mmol) and 5.0 mL of 1,4-dioxane were added into a dry 50 mL ampoule equipped with a stirrer. The ampoule was then degassed by freeze-pump-thaw technique three times in the liquid nitrogen bath, and then sealed under vacuum. The system was heated to 70 °C for 1h, and then gradually heated to 220 °C and kept at this temperature for 48 h. After cooling to room temperature, the product was isolated and washed successively by acetone, methanol and THF, and finally extracted with THF in a Soxhlet apparatus for 24 h. The obtained white solid was dried at 100 °C in vacuo to constant weight (Yield: 77% for PFcMOP ; 64% for CFcMOP).

Photocatalytic Activity Measurements

The photocatalytic activities of the MOPs were evaluated by degradation of methylene blue (MB) under visible light irradiation at room temperature. The visible light source was a 300 W xenon lamp equipped with an optical cut off filter (λ ≥ 420 nm). Typically, 3 mg of the photocatalyst powder was dispersed into 5 mL organic pollutant solution with a concentration of 10 mg L^{-1}. Prior to light illumination, the suspension was magnetically stirred for 1 h in the dark to reach an adsorption–desorption equilibrium, and then At the given intervals, 0.2 mL of aliquot was extracted

and filtered to remove the photocatalyst. After that, the resulting supernatants were analyzed by monitoring the absorbance spectra on a UV–vis spectrophotometer (TU-1901).

Photodegradation mechanism study was conducted by preparation 5 mL $CuSO_4$ solution (0.48 mmol L^{-1}),5 mL EDTA-2Na solution (5 mmol L^{-1}), 5 mL L-His (1.6 mmol L^{-1}) and 5 mL tert-butanol (1 mmol L^{-1}), respectively, as activity species quenching agent to 3 mg MOPs in MB aqueous solution (10 mg L^{-1}) before visible light source irradiation.

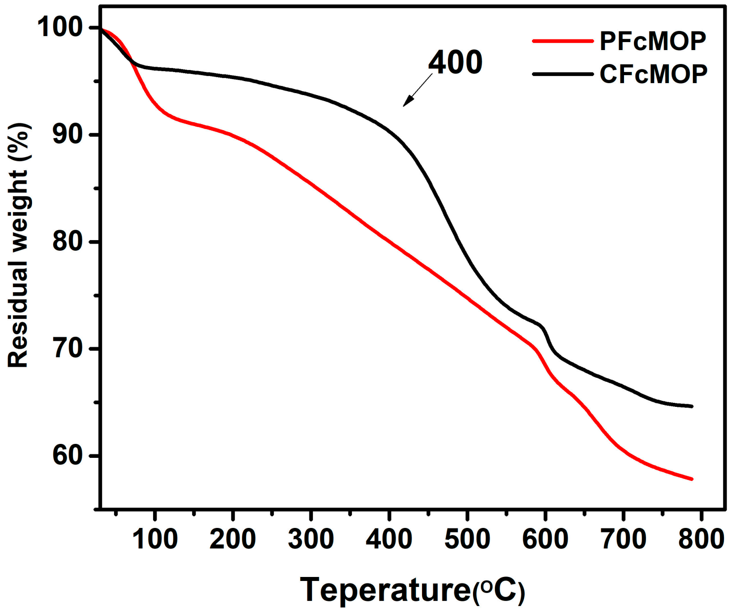

Figure S1. TGA curves of the two ferrocenyl microporous polymer networks.

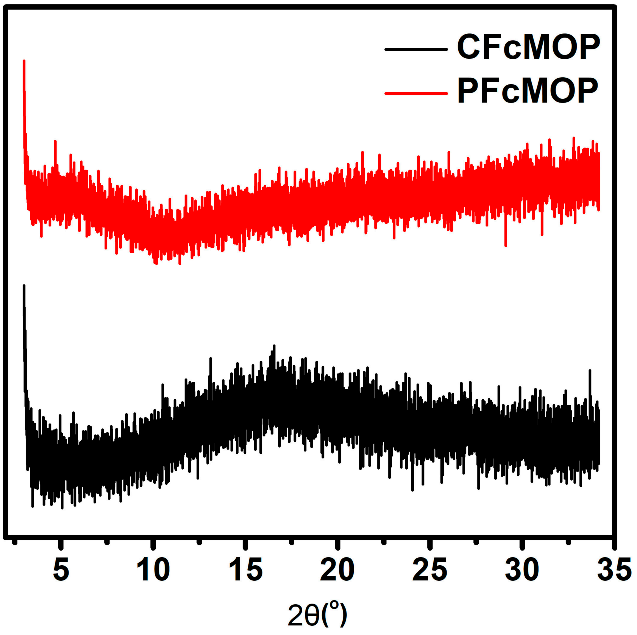

Figure S2. PXRD curves of the two ferrocenyl microporous polymer networks.

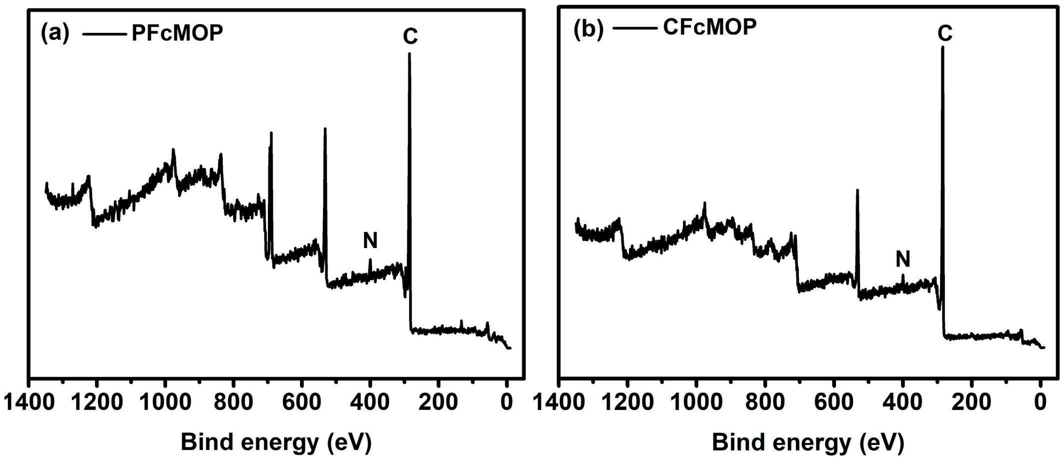

Figure S3. XPS curves of the two ferrocenyl microporous polymer networks.

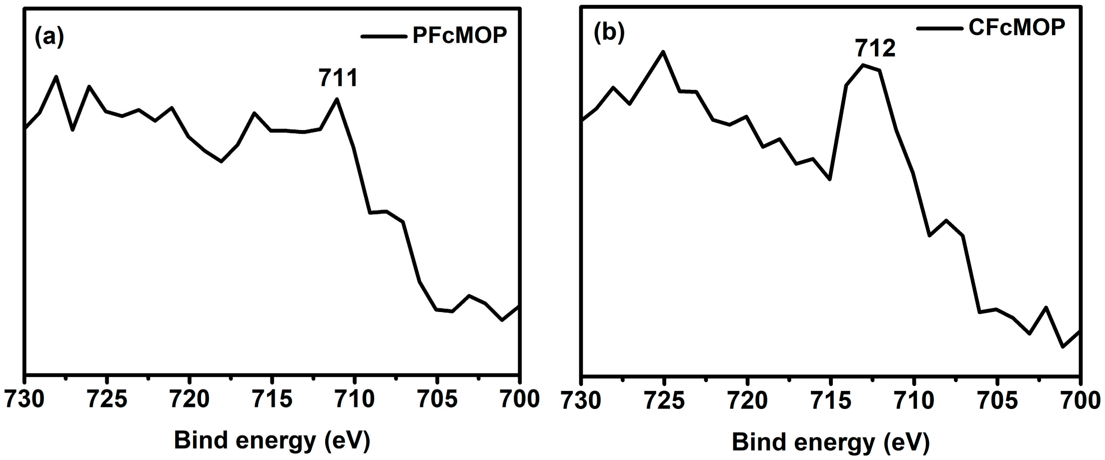

Figure S4. XPS curves of Fe element in the two ferrocenyl microporous polymer networks.

Table S1. C, Fe, and N element percent of PFcMOP and CFcMOP

Elemental content(%)		C	N	Fe
PFcMOP	Experimental value	91.30	3.17	4.91
	Theoretical value	86.95	8.69	4.34
CFcMOP	Experimental value	92.12	3.24	4.64
	Theoretical value	92.30	5.12	2.56
